# A microarray analysis of sex- and gonad-biased gene expression in the zebrafish: Evidence for masculinization of the transcriptome

**DOI:** 10.1186/1471-2164-10-579

**Published:** 2009-12-03

**Authors:** Clayton M Small, Ginger E Carney, Qianxing Mo, Marina Vannucci, Adam G Jones

**Affiliations:** 1 Department of Biology, Texas A&M University, College Station, TX, USA; 2 The Center for Environmental and Rural Health, Texas A&M University, College Station, TX, USA; 3 Department of Statistics, Rice University, Houston, TX, USA; 4 The Department of Epidemiology and Biostatistics, Memorial Sloan-Kettering Cancer Center, New York, NY, USA

## Abstract

**Background:**

In many taxa, males and females are very distinct phenotypically, and these differences often reflect divergent selective pressures acting on the sexes. Phenotypic sexual dimorphism almost certainly reflects differing patterns of gene expression between the sexes, and microarray studies have documented widespread sexually dimorphic gene expression. Although the evolutionary significance of sexual dimorphism in gene expression remains unresolved, these studies have led to the formulation of a hypothesis that male-driven evolution has resulted in the masculinization of animal transcriptomes. Here we use a microarray assessment of sex- and gonad-biased gene expression to test this hypothesis in zebrafish.

**Results:**

By using zebrafish Affymetrix microarrays to compare gene expression patterns in male and female somatic and gonadal tissues, we identified a large number of genes (5899) demonstrating differences in transcript abundance between male and female *Danio rerio*. Under conservative statistical significance criteria, all sex-biases in gene expression were due to differences between testes and ovaries. Male-enriched genes were more abundant than female-enriched genes, and expression bias for male-enriched genes was greater in magnitude than that for female-enriched genes. We also identified a large number of genes demonstrating elevated transcript abundance in testes and ovaries relative to male body and female body, respectively.

**Conclusion:**

Overall our results support the hypothesis that male-biased evolutionary pressures have resulted in male-biased patterns of gene expression. Interestingly, our results seem to be at odds with a handful of other microarray-based studies of sex-specific gene expression patterns in zebrafish. However, ours was the only study designed to address this specific hypothesis, and major methodological differences among studies could explain the discrepancies. Regardless, all of these studies agree that transcriptomic sex differences in *D. rerio *are widespread despite the apparent absence of heterogamety. These differences likely make important contributions to phenotypic sexual dimorphism in adult zebrafish; thus, from an evolutionary standpoint, the precise roles of sex-specific selection and sexual conflict in the evolution of sexually dimorphic gene expression are very important. The results of our study and others like it set the stage for further work aimed at directly addressing this exciting issue in comparative genomics.

## Background

The evolution of phenotypic differences between males and females, which are often spectacular, has been a subject of intense scrutiny since Darwin [1]. Several well-studied, often-integrated forms of sexual dimorphism include morphological [1], behavioral [2], and physiological [3] differences. Clearly, the evolutionary mechanisms ultimately responsible for sexual dimorphism (i.e., sexual selection [4], sex-specific ecological selection [4], and sexual conflict [5]) are of great interest. However, a complete understanding of these processes is impossible without knowledge of the proximate genetic and genomic underpinnings of sex-limited phenotypes.

Several proximate mechanisms can account for the phenotypic differences between males and females. For instance, fixed genetic differences between males and females via heteromorphic sex chromosomes [6] or a sex-determination locus provide one basis for sexual dimorphism. In this case, the two sexes possess partially distinct genomes. However, phenotypic sexual dimorphism may also be mediated by sex differences in gene *expression *when a key transcript differs in abundance between males and females. These two mechanisms are by no means mutually exclusive, as sex-specific aspects of the genome result in downstream sex differences in gene expression at sex-shared loci, especially when the original sex-unique genes are highly pleiotropic (e.g. they affect multiple developmental pathways). Sexes need not have distinct genomes for sexual dimorphism to exist, however, because species characterized by environmental sex determination nevertheless maintain a considerable degree of sex-based phenotypic differentiation with respect to primary and often secondary sexual traits [7-9]. In these cases of non-genetic sex determination, sex differences in gene expression are obviously important sources of sexual differentiation and dimorphism.

Some interesting gene expression patterns with regard to sex have been reported over the past several years, initially in *Drosophila melanogaster *and later in other taxa (see a recent review of sex-biased gene expression by Ellegren and Parsch [10]). One observation is that of those genes that demonstrate sex-biases in expression level, more tend to be male-enriched than female-enriched [11-15] (but see [12,16]). This high level of observed sexual dimorphism in gene expression is mostly attributable to differences between testis and ovary [11]. Furthermore, male-enriched genes are more divergent in their expression levels among species than are female-enriched or sex-unbiased genes [17]. These patterns, in addition to the discovery that male-enriched genes also demonstrate faster rates of DNA sequence evolution relative to female-enriched and sex-unbiased genes [18], have been interpreted as a general signature of stronger sexual selection on males. This "male sex drive" hypothesis, formally proposed by Singh and Kulathinal [19], is consistent with findings across several animal taxa. However, additional independent tests of this hypothesis should be carried out before it is accepted as a general pattern of evolution.

In this study we take advantage of the zebrafish as a model of vertebrate reproduction to test predictions under the male sex drive hypothesis. Environment, hormones, and genetic components likely influence sex differentiation in *Danio rerio*, but the precise roles and interactions of these factors with respect to reproductive development remain unclear [20,21]. Takahashi [22] originally described zebrafish gonad differentiation as a transition from a two-weeks-post-fertilization ovary-like precursor to either the mature ovary or the highly differentiated testis. This transition from ovary-like precursor to testis in males is mediated by oocyte apoptosis, which is generally complete by 29 days post-hatching [23]. More recently it has been shown that some male zebrafish exhibit few ovary-like features and lack ovary-typical gene expression during gonadal development [24]. In fact, males vary dramatically in the developmental timing and abundance of ovarian features (genetic and morphological) leading up to testis formation, and there is even substantial variation within sibling groups [21]. Sexual maturity in zebrafish is attained well after gonad differentiation, and usually is complete when individuals reach 23-25 mm standard length (approximately 75 days post-hatching for domesticated strains) [25].

One advantage to zebrafish is that Affymetrix GeneChip^® ^technology is readily available, permitting the assessment of large-scale patterns of expression in adults and their gonads. The Zebrafish Genome Array design is based on sequence information from RefSeq (July 2003), GenBank (release 136.0, June 2003), dbEST (July 2003), and UniGene (Build 54, June 2003). With approximately 14,900 transcripts represented on the array, this technology can provide a representative sample of sex differences in gene expression patterns. Our goal was to compare gene expression patterns between testes and ovaries as well as between male and female somatic tissue. A collateral benefit to these comparisons was that we were also able to identify genes within each sex that were up- or downregulated in the gonads. Under the male sex drive hypothesis, we expected more genes upregulated in males relative to females. We predicted many of these genes to be gonad specific, but also expected to find some genes expressed at different levels in the somatic tissues of males compared to females.

While our study is the first to explicitly address the male sex drive hypothesis in *Danio rerio*, several recently published microarray studies of gene expression in zebrafish have addressed aspects of sexually dimorphic gene expression and gonad specific expression patterns. In general these studies have revealed that the quantities of particular transcripts often differ significantly in adult males and females, at the level of the whole body [26], the gonads [27,28], the brain [28,29], the liver [30], and other tissues [28]. However, these studies do not necessarily agree with ours on all points related to patterns relevant to the evolution of sex-biased gene expression in zebrafish, so we will return to this topic in the discussion.

## Methods

### Affymetrix GeneChip^® ^preparation

We allowed eight mating pairs of wild-type (AB laboratory strain) *Danio rerio *to spawn under controlled laboratory conditions and subsequently separated the sexes for a period of 5 days to prevent re-mating and standardize reproductive cycles. To minimize inter-individual differences among the fish, all subjects were full siblings, between 4 and 12 months old. After sacrificing each individual by ice bath euthanasia, we quickly excised all testicular tissue from males and all ovarian tissue from females. All methods were approved by Texas A&M University's Institutional Animal Care and Use Committee (AUP2005-76). Tissues were flash-frozen in TRIzol^® ^Reagent (Invitrogen), and total RNA isolation was performed in accordance with the manufacturer's guidelines. Following quantification and quality assessment, total RNA samples from 3 testis pairs, 3 male bodies, 3 ovary pairs, and 3 female bodies were sent to the University of Kentucky Microarray Core Facility for cRNA labeling and hybridization to 12 GeneChips^® ^using standard Affymetrix protocols (described in the GeneChip^® ^Expression Analysis Technical Manual). Briefly, total RNA was reversed transcribed, followed by production of biotinylated cRNA. After a fragmentation step the biotinylated cRNA was hybridized to the arrays for a period of 16 hours. The samples were then stained with streptavidin phycoerythrin and amplified using a biotinylated anti-streptavidin antibody prior to scanning.

### Absolute expression analyses

The GeneChip^® ^Zebrafish Genome Array contains ~15,500 probe sets, each set consisting of 16 adjacent but non-overlapping probe pairs. These probe pairs are 25 bases long, each pair containing one probe (*PM*) that perfectly matches the target transcript and another probe (*MM*) that mismatches the target sequence at a single base pair. The presence of a mismatch probe is intended to control for background noise caused by hybridization of non-target molecules. To convert array image information into transcript abundance values, we employed four different "absolute expression analysis" algorithms. Each of these analysis methods was used to generate a distinct dataset from a given chip image file. We applied standard normalization procedures to raw data prior to analysis, as suggested by each respective program manual. Normalized expression values for all absolute analyses across all experimental replicates, along with other pertinent microarray details, have been deposited into the NCBI Gene Expression Omnibus (GEO) under accession number GSE14979.

#### GCOS

The algorithm implemented in the GCOS software package (Affymetrix), uses the one-step Tukey's biweight mean of *PM*_*i *_- *CT*_*i *_across *i *probe pairs, where *PM *is the intensity of the perfect match probe cell, and *CT *is the "contrast value" [31,32]. *CT *is most often equal to *MM *(the intensity value of the mismatch probe cell), but if many probe pairs within a set demonstrate *MM *values larger than their corresponding *PM *values, an adjusted value is used for *CT *to eliminate the computation of negative expression values [33]. This algorithm is therefore a simple calculation based on subtracting background noise from the putative "true signal."

#### GC-RMA

We also used the GC-RMA (GC Robust Multi-Array Analysis) algorithm, as implemented in the microarray analysis software package GeneSpring GX 7.3.1 (Agilent). The GC-RMA algorithm is based on a linear additive model, and thus considers all arrays in a given dataset when estimating expression values for each chip, unlike the GCOS algorithm. The basic linear model is described by Wu et al. [34], and assumes that *Y*_*gij *_= *O*_*gij *_+ *N*_*gij *_+ *S*_*gij*_, where *Y*_*gij *_is the *PM *intensity value for probe *j *in probe set *g *on array *i*. *O*_*gij *_is the corresponding "optical noise" due to laser scanning errors, *N*_*gij *_is the corresponding "non-specific binding noise," and *S*_*gij *_is a quantity proportional to the actual abundance of target transcript in a sample (which allows for estimation of the "true" expression value). The GC-RMA algorithm uses many parameters from the observed data in all arrays to estimate components of *N*_*gij *_and *S*_*gij*_, then it fits the model to calculate expression values [34].

#### PM-MM, PM-Only

Two additional model-based approaches, available in the analysis package dChip [35,36], were also used to generate expression values. The PM-MM model assumes that for every probe set in a group of *i *arrays, *PM*_*ij *_- *MM*_*ij *_= *θ*_*i *_*φ*_*j *_+ *ε*_*ij*_, where *PM*_*ij *_and *MM*_*ij *_are the perfect match and mismatch intensities for probe pair *j *in array *i*, *θ*_*i *_is the expression index for the probe set in array *i *(the value of interest), *φ*_*j *_is a coefficient that represents the relationship between probe pair *j *cell intensities and actual target concentration, and *ε*_*ij *_is the model's error term [33,35,36]. Similar to GC-RMA, the PM-MM algorithm uses information from all chips in a dataset, and then the model is fit to estimate the expression value for each probe set on each chip. The PM-Only algorithm is similar to PM-MM, but the mismatch intensities are completely ignored in the model: *PM*_*ij *_= *θ*_*i *_*φ*_*j*_+ *ε*_*ij*_. This alternative model was created to avoid the occasional calculation of negative expression values when *MM *probe intensities are high compared to *PM *intensities [35,36].

### Comparative expression analyses

To compare absolute expression values between different treatment groups, detect differential transcript levels, and estimate fold changes, we conducted standard t-tests using the Cyber-T web interface [37]. This approach yielded 4 sets (one per absolute expression algorithm) of results for each of the following comparisons: male body vs. female body, testis vs. ovary, testis vs. male body, and ovary vs. female body. To control for the statistical problem of performing ~15,000 t-tests per comparison, we set a false discovery rate (FDR) of 0.05, as described by Benjamini and Hochberg [38], for each analysis. To decide whether a gene for a given comparison was to be considered "differentially expressed," we adopted a "strict consensus" criterion wherein the gene was required to demonstrate a significant FDR-adjusted p-value across all 4 absolute analysis datasets. This procedure is conservative, but justifiable in the name of controlling for false positives.

### Real-time PCR

We used the remaining 5 male and 5 female zebrafish samples to conduct independent tests of expression bias for seven genes identified as differentially expressed by our microarray analyses. Within each of the testis-upregulated, male-enriched, and female-enriched categories we randomly chose two of the top ten most upregulated genes. We were able amplify a gene-specific PCR product for only one of the chosen male-enriched transcripts (probe set 15637.1.S1_at). Within the ovary-upregulated category, we randomly chose two of the top 200 most upregulated genes, in order to assess the accuracy of microarray results for genes demonstrating less striking differences in expression. For each sample the same quantity of total RNA (1 μg) was reverse transcribed into cDNA using the Superscript^® ^First Strand Synthesis Kit (Invitrogen).

We performed real-time PCR using the SYBR^® ^Green PCR Mastermix (Invitrogen) and 2 μl of cDNA template. Reactions were run on an ABI 7700 real-time PCR apparatus (Applied Biosystems) using default analysis settings. Each individual reaction was performed in triplicate, and no-template controls were included for each primer pair to confirm amplification specificity. A dilution series including 5 different template concentrations was employed to facilitate the Relative Standard Curve Method (Applied Biosystems) for estimating relative mRNA levels. Primer sequences for target genes were designed using Primer Express^® ^3.0 (Applied Biosystems) and are available upon request. Two sets of control primers (suggested in Tang et al. [39]) were used to normalize the abundance of cDNA in each reaction. *EF1α *was used in the gonad-body comparisons, and *Rpl13α *was used in the male-female comparisons. For each comparison we calculated a 95% confidence interval about mean fold change, based on the expression level estimates across the 5 experimental replicates.

## Results

### Sex-biased gene expression

To assess the extent of sex-biased gene expression in *Danio rerio *we compared male body to female body transcript levels, and we performed a separate testis-ovary comparison. This effectively allowed us to isolate the proportion of sex-biased gene expression attributable to differences between male and female gonads. To avoid any confusion about references to the different gene expression categories, Table 1 outlines the relevant terminology, to which we hereafter adhere. Based on our expression bias criteria, 5899 out of 15502 probe sets (38%) represented on the Affymetrix zebrafish GeneChip^® ^demonstrated statistical testis-ovary differences, across all 4 absolute expression analyses, in transcript abundance. 1737 probe sets yielded an insufficient signal in all ovary and testis replicates. Of the 5899 sex-biased genes, 3387 were positively biased in males ("male-enriched"), and 2512 were positively biased in females ("female-enriched") (Table 2), consistent with the overall direction of sex-biased gene expression documented in other taxa [11-14]. Also represented in Table 2 are the numbers of sex-biased genes corresponding to increasingly stringent fold change criteria. From this information it is clear that the direction of sex-biased gene expression remains robust, even when genes demonstrating small sex differences in expression are not considered. Additional Files 1 and 2 contain lists of all male- and female-enriched genes, respectively. Other zebrafish studies have detected male- and female-enriched genes via comparison of testis and ovary [27,28]. We selected five male-enriched and five female-enriched genes from Santos et al. [27] and from Sreenivasan et al. [28] to confirm that these 20 genes fall into the same expression categories in our study (see "male-enriched" and "female-enriched" sections of Table 3). We selected these genes because they ranked at the top of their respective lists in regard to the magnitude of expression bias. As Table 3 indicates, 18 out of these 20 major sex-biased genes from references 27 and 28 are also among our list of sex-biased genes.

**Table 1:** Terms used to describe gene expression categories in this study

Term	Explanation
Male-enriched	Genes demonstrating greater transcript abundance in the testes relative to the ovaries.

Female-enriched	Genes demonstrating greater transcript abundance in the ovaries relative to the testes.

Testis-upregulated	Genes demonstrating greater transcript abundance in the testes relative to the male body (from which the testes have been removed).

Ovary-upregulated	Genes demonstrating greater transcript abundance in the ovaries relative to the female body (from which the ovaries have been removed).

Terms used to describe relevant categories of gene expression. Statistically significant male- and female-enriched genes in our study correspond to differences between testis and ovary only.

**Table 2:** Expression bias and increasing fold change threshold

Expression bias class	No fold threshold	≥ 1.5 fold	≥ 2 fold	≥ 4 fold	≥ 6 fold
Male-enriched	3387	3219	2576	1196	728

Female-enriched	2512	2281	1684	664	413

Testis-upregulated	3002	2824	2159	925	554

Ovary-upregulated	981	842	426	0	0

Number of sex- and gonad-biased genes (strict consensus FDR = 0.05) under increasing fold change thresholds. As the fold change criterion becomes more stringent, fewer genes are deemed differentially expressed, but the male-biased patterns remains consistent. The numbers above reflect genes that satisfy the indicated fold change thresholds across all four absolute expression analyses.

**Table 3:** An across-study comparison of sex- and gonad-biased gene expression in zebrafish

Gene Name, EST accession number (if applicable)	Reference	Fold Rank (This Study)	GCOS Fold	GC-RMA Fold	PMMM Fold	PM Only Fold
***Male-enriched Genes***						

*anti-Mullerian hormone (amh)*	27	18	328.39	154.95	78.09	80.60

*cyclin G2 (ccng2)*	27	690	328.39	11.78	7.74	8.85

*heat shock cognate 70-kd protein (hsp70)*	27	-	3.64	2.63	2.20	2.63

similar to *septin 4 (sept4)*	27	2	608.87	673.38	48.17	364.57

*tubulin, alpha 7 like (tuba7l)*	27	19	235.72	985.76	46.06	66.53

similar to *tektin 1*, CO352798	28	3	484.87	681.64	49.65	196.92

*dynein, axonemal, intermediate polypeptide 1 (dnai1)*, CO355627	28	45	144.47	186.74	27.69	50.98

similar to *human AKAP-associated sperm protein*, CO353327	28	83	58.05	181.15	22.55	44.38

*piwi-like 1 (Drosophila) (piwil1)*, CO354057	28	1261	6.81	8.28	5.12	5.01

similar to *testis-specific-A-kinase-anchoring protein*, CO354405	28	4	409.79	416.74	58.48	174.20

***Female-enriched Genes***						

*transmembrane phosphatase with tensin homology (tpte)*	27	1084	3.34	4.50	3.30	3.48

*RNA binding protein with multiple splicing 2 (rbpms2)*	27	216	26.94	48.76	14.73	20.47

*connexin 44.2 (cx44.2)*	27	139	66.33	198.28	31.92	54.72

*SRY-box containing gene 11b (sox11b)*	27	187	30.21	79.53	22.06	23.13

*cyclin B2 (ccnb2)*	27	284	14.58	28.39	12.39	13.34

similar to *egg envelope glycoprotein*, CO350790	28	132	62.31	190.67	53.93	59.02

*flap structure-specific endonuclease 1 (fen1)*, EV603088	28	-	1.11	1.17	1.10	1.10

hypothetical protein LOC556628, CO350423	28	30	273.38	1679.77	110.57	156.60

*B-cell translocation gene 4 (btg4)*, CO349959	28	75	168.79	416.47	78.66	168.05

similar to *transcription factor IIIA*, CO349799	28	138	58.00	147.42	40.73	60.90

***Testis-upregulated Genes***						

zgc:162225, CO352964	42	139	282.01	121.35	9.68	33.48

*WD repeat-containing protein 69*, CO355324	42	82	59.34	175.15	21.17	41.71

zgc:158652, CO353149	42	-	113.93	392.11	30.49	42.58

zgc:112008, CO352835	42	176	28.11	111.65	13.66	24.40

similar to CG14551-PA, CO352954	42	9	301.99	280.30	46.03	207.76

hypothetical protein LOC558005, CO355049	28	147	69.83	220.63	24.25	14.03

unknown transcript, CO355999	28	383	12.96	9.27	12.50	56.80

hypothetical protein LOC100003104, CO353145	28	13	165.59	1010.46	42.16	81.04

similar to *polyprotein*, CO355597	28	63	51.37	131.92	47.53	48.39

similar to *tektin 1*, CO353325	28	12	167.65	848.47	57.56	69.06

***Ovary-upregulated Genes***						

hypothetical protein LOC100001369, CO350972	42	-	3.45	3.29	2.22	2.44

hypothetical protein LOC555929, CO351149	42	-	2.66	5.13	2.19	3.21

similar to *novel rhamnose binding lectin*, CO350303	42	-	1.17	1.21	1.14	1.20

unknown transcript, CO350393	42	-	2.03	2.79	1.88	1.97

similar to *egg envelope glycoprotein*, CO350790	28	-	1.37	1.92	1.48	1.52

wu:fi40a06, CO349940	28	-	1.26	1.93	1.48	1.49

*DEAD (Asp-Glu-Ala-Asp) box polypeptide 56*, CO354027	28	810	2.38	2.47	1.60	1.58

hypothetical protein LOC447813, CO350110	28	-	2.02	1.76	1.02	1.02

clone MGC:55720, CO350755	28	909	2.13	2.37	1.35	1.35

*retinol saturase like (retsatl)*, CO350808	28	-	1.75	2.49	1.79	1.85

List of sex- and gonad-biased genes identified by other recent zebrafish studies [27,28,42]. The sex-biased genes are based on testis-ovary comparisons, as in our study. These genes were chosen from the above studies based on reportedly high expression bias. We screened our lists of differentially expressed genes to assess agreement with the other studies. The "fold rank" is the position each gene occupies in our lists, based on the mean of rank across the four absolute expression comparisons. *sept4*, for example, is the gene demonstrating the second-highest male-enriched expression (out of 3387 total male-enriched genes). No rank is listed if the gene failed to pass our "strict consensus" statistical criteria (see Methods). Also listed are fold change estimates corresponding to each of the four absolute expression analyses.

It is important to note that we detected no gene expression biases between male and female body tissue under our strict criteria for significance (1574 probe sets demonstrated an insufficient signal in all male body and female body replicates). If we relax our criteria by allowing statistical significance in any one of the four analysis algorithms (as opposed to all four) to constitute evidence of differential expression, then we find 112 genes that are differentially expressed between male and female body tissue. This list of putative sexually dimorphic genes is included as supplementary information (Additional File 3), but these genes are not considered in further analyses within this study. Indeed, other microarray studies of zebrafish have demonstrated sex differences in isolated organs such as the liver [30] and the brain [29], but according to our results, the vast majority of sex-biases in zebrafish gene expression are due to transcriptomic differences between testis and ovary. This observation is consistent with studies of other taxa in which tissue-specific contributions to sex-biased gene expression have been parsed out [11,14,40].

**Figure 1:**
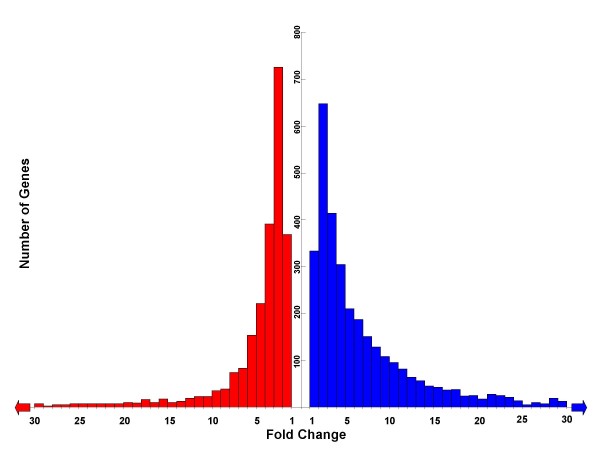
**Expression bias for male-enriched genes is greater than expression bias for female-enriched genes**. Histogram showing the distributions of fold change values for female-enriched (red) and male-enriched (blue) genes. Recall that our differential expression criteria revealed 2512 female-enriched and 3387 male-enriched genes. Each observation represented in this graph is a mean across four fold change values, corresponding to the four different absolute expression analyses. Arrows at x-axis termini represent distribution tails, which are not shown. These tails (approximately 200 observations each) were omitted for ease of graphical representation, and their absence does not affect the interpretation of the histogram. Comparison of the two distributions reveals that male-enriched genes are more frequent at higher fold change intervals, relative to female-enriched genes, and a Mann-Whitney U test formally confirms higher fold change values for male-enriched genes (p < 0.001).

To further examine whether the overall magnitude of sex-biased gene expression in zebrafish is greater for male-enriched genes, we compared fold change values of male-enriched genes to those of female-enriched genes. For each gene, the mean fold change estimate across all four absolute expression analysis estimates (GCOS, GC-RMA, PMMM, and PM-only) was used to represent the magnitude of expression bias. The male-enriched and female-enriched distributions of this variable are significantly different (Mann-Whitney *U *Test, p < 0.001), the male-enriched fold change values being greater in magnitude. Frequency distributions of male- and female-enriched genes are represented graphically in a mirrored histogram (Fig. 1). Based on Fig. 1, it is evident that the male-enriched gene distribution includes more "high fold change" observations than the female-enriched distribution.

### Gonad-biased gene expression

It might be argued that transcripts more abundant in an organism's gonads relative to its body correspond to genes especially relevant to reproduction. In light of this, we thought it would be informative and useful to identify putative reproductive genes in *Danio rerio*. According to our criteria for differential expression, 3002 genes represented on the array were upregulated in the testes, and 2338 were downregulated (1297 probe sets yielded an insufficient signal in all testis and male body samples). 981 genes were upregulated in the ovaries, and 1399 were downregulated (1917 probe sets produced an insufficient signal in all ovary and female body samples). The numbers of differentially expressed genes decline as one imposes more stringent fold-change criteria (Table 2), and it appears that ovary-upregulated genes demonstrating high fold changes are scarce, relative to high-fold testis-upregulated genes. Complete lists of testis- and ovary-upregulated genes are included as Additional Files 4 and 5, respectively. Our results indicate that male, compared to female, zebrafish possess many more genes whose expression is elevated in gonads.

To identify which testis- and ovary-upregulated genes demonstrated the highest gonad specificity, we ranked each gene based on its average fold change ranking across each absolute expression analysis dataset. Tables 4 and 5 report the 15 highest ranking testis- and ovary-upregulated genes, respectively. For some of the genes corresponding to known or predicted *Danio rerio *mRNAs, functional annotation information is available. In some cases (Table 4) this information confirms the presumed reproductive functions of these genes. The testis-enriched gene *odf3l*, for example, codes for a structural protein (SHIPPO 1) associated with the sperm flagellum [41], and may therefore be of relevance with respect to sperm competition. For the most part, however, it is difficult to speculate on the actions of gene products that remain largely uncharacterized.

**Table 4:** Top 15 ranked testis-upregulated genes within male zebrafish

GenBank acc. #	GenBank reference mRNA sequence	GCOS fold	GC-RMA fold	PM fold	PMMM fold
NM_001082815	similar to *septin 4 *(*sept4*)	590	1162	47	161

NM_212833	zgc:56699	404	1501	40	252

BI709397	unknown. No significant BLAST hits.	254	1026	51	96

BI709397	unknown. No significant BLAST hits.	412	451	36	331

NM_199958	***outer dense fiber of sperm tail gene 3-like *(*odf3l*)**	**162**	**787**	**71**	**97**

NM_212806	*cytochrome P450, family 17, subfamily A, polypeptide 1 *(*cyp17a1*)	157	368	92	184

NM_131057	***vasa homolog *(germ line development)**	**287**	**558**	**40**	**145**

NM_001100021	UPF0722 protein, *C11orf88 *homolog	146	541	73	101

XM_692188	similar to CG14551 CG14551-PA	302	280	46	208

NM_001002357	zgc: 92129	349	1785	69	61

NM_001118894	***synaptonemal complex protein 1 *(*sycp1*)**	**452**	**191**	**62**	**191**

NM_001007397	zgc:101797	168	848	58	69

XM_001342700	similar to predicted protein (LOC100003104)	166	1010	42	81

XM_692362	wu:fj98c04	187	401	34	185

NM_001089414	hypothetical protein zgc:162591	203	337	47	95

Fifteen highest ranking testis-upregulated genes (of 3002 total), determined by the mean of all four fold change rank values for each of the absolute expression analyses. Basic annotation is represented by a top MegaBLAST hit for each GeneChip^® ^probe set sequence, obtained by a search of the GenBank reference mRNA database. Any supplementary functional annotation information is included if available. E-values for the above BLAST searches are all 0.0, except for *sept4 *(5 e-65) and *cyp17a1 *(1 e-123). Several of the probe sets listed here lack any information with respect to a described mRNA counterpart, and many correspond to hypothetical protein-coding transcripts. Three of the well-annotated transcripts (in bold text), appear to be reproduction-related.

**Table 5:** Top 15 ranked ovary-upregulated genes within female zebrafish

GenBank acc. #	GenBank reference mRNA sequence	GCOS fold	GC-RMA fold	PM fold	PMMM fold
XR_044724	zgc:109744	5.2	9.8	3.4	5.5

NM_001123299	similar to CG14692-PA	5.0	8.8	3.8	4.4

XM_678859	similar to *tripartite motif protein 33*	4.7	9.6	3.4	5.5

NM_001003609	*microtubule associated serine/threonine kinase-like *(*mastl*) [associated with amino acid phosphorylation]	4.4	7.6	3.6	4.0

BM957577	unknown. No significant BLAST hits.	4.1	7.5	3.4	3.9

NM_200329	*globoside alpha-1,3-N acetylgalactosaminyltransferase 1-like 1 *(*gbgt1l1*) [homologous to *mammalian ABO transferase A*]	5.0	5.6	3.6	3.8

XM_001920491	similar to *Tudor domain-containing protein 6 *(Antigen NY-CO-45) (Cancer/testis antigen 41.2) (CT41.2)	4.9	7.2	2.6	4.6

NM_001017680	*F-box protein 16 *(*fbxo16*)	4.9	6.2	3.1	3.3

NM_001123056	zgc:172124 [homologous to *protein kinase C, eta*]	4.4	9.0	2.6	4.3

NM_001098186	*suppressor of variegation 4-20 homolog 2 *(*Drosophila*) (*suv420h2*)	3.6	12.2	3.9	5.4

NM_001020771	zgc:112481	4.3	5.3	3.1	4.0

XM_001339628	*jumonji domain containing 2A-like *(*jmjd2al*)	4.4	6.3	2.9	3.3

NM_001002551	*non-SMC element 1 homolog *(*S. cerevisiae*) (*nsmce1*)	4.1	6.1	2.6	4.3

NM_001077170	im:7162391, *nephrocystin-1*	3.9	6.5	2.8	3.3

NM_001100948	*granulito*	3.9	5.3	3.2	3.4

Fifteen highest ranking ovary-upregulated genes (of 981 total), determined by the mean of all four fold change rank values for each of the absolute expression analyses. Basic annotation is represented by a top MegaBLAST hit for each GeneChip^® ^probe set sequence, obtained by a search of the GenBank reference mRNA database. Any supplementary functional annotation information is included if available. E-values for the above BLAST searches are all 0.0, except for *nsmce1 *(2 e-152). Several of the probe sets listed here lack any information with respect to a described mRNA counterpart, and many correspond to hypothetical protein-coding transcripts.

Other studies have identified genes upregulated in or specific to zebrafish gonads, based on various methods and expression criteria [28,42,43]. We selected five testis-upregulated and four ovary-upregulated genes from Li et al. [42], and five testis-upregulated and six ovary-upregulated genes from Sreenivasan et al. [28] to confirm that these 20 genes fall into the same expression categories in our study (see "testis-upregulated" and "ovary-upregulated" sections of Table 3). We selected these genes because they ranked at the top of their respective lists in regard to the magnitude of expression bias. While our study agrees with these other two studies quite well in terms of testis-upregulated genes, there is rather poor agreement over ovary-upregulated genes.

**Figure 2:**
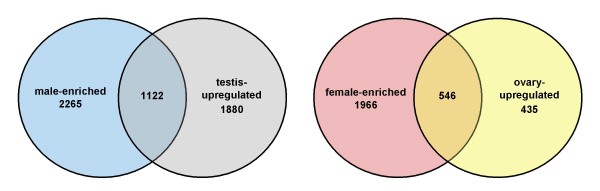
**Overlap of sex- and gonad-biased gene expression**. Male (left) and female (right) Venn diagrams, demonstrating the proportion of genes that fall into both sex- and gonad-biased expression categories. These numbers are based on a "strict consensus" FDR = 0.05, and no fold change threshold. Roughly 33% of male-enriched genes are also significantly testis-upregulated, whereas approximately 22% of female-enriched genes are also significantly ovary-upregulated.

There is a large categorical overlap with respect to sex- and gonad-biased gene expression (Fig. 2). Approximately 27% of the genes that were identified as being either male-enriched or testis-upregulated intersect. This dual categorical identity also exists for ~23% of genes that are either female-enriched or ovary-upregulated. In general, a substantial proportion of genes upregulated in the gonads of each sex are also expressed differentially between male and females.

### Validation of microarray expression measurement

We used real-time PCR to confirm transcription bias in a subset of genes, representing the four different microarray expression bias categories relevant in this study (See Methods for details). Seven genes (two ovary-upregulated, two testis-upregulated, two female-enriched, and one male-enriched) were selected based on high fold change rank within each class and amenability to successful PCR amplification. A summary of the validation experiment is shown in Table 6, and raw expression values and statistical tests are reported in Additional File 6. We calculated 95% confidence intervals for transcript abundance, and hence fold change, (*N *= 4 - 5) in each case. The confidence intervals are extremely wide for many of the genes, probably due to real variation among individual fish and a small sample size. Nevertheless, each independent test confirmed a significant expression bias in the expected direction, and confidence interval width seems to scale with variation in array fold change estimates across the four different absolute expression analyses.

**Table 6:** Real-time PCR expression confirmation

Gene class	Gene Name/ GenBank acc. #	Rank	GCOS fold	GC-RMA fold	PM fold	PMMM fold	qPCR 95% CI
Ovary-upregulated	*casp3a * NM_131887	121	3.0	4.7	2.4	2.7	4.6 -15

Ovary-upregulated	zgc:92067 NM_001002377	187	2.4	6.1	2.5	2.7	13 - 57

Testis-upregulated	*sept4 * NM_001082815	1	590	1162	47	161	189 - 518

Testis-upregulated	zgc:92129 NM_001002357	10	349	1785	69	61	740 - 3665

Male- enriched	fx05c05.x1 BM571726	4	810	545	38	283	189 - 488

Female-enriched	wu:fd20g04 XM_001334198	1	458	2190	246	551	1982 - >9999

Female-enriched	wu:fd14c01 XM_677844	2	572	1458	174	479	609 - 3246

Expression levels of sex- and gonad-biased zebrafish genes, as confirmed by quantitative real-time PCR. Included are each gene's expression bias category, GenBank identifier and accession number, within-category expression rank, four microarray fold change estimates based on different absolute expression analyses, and qPCR 95% confidence interval for fold change. Some of the confidence intervals are quite wide, but in every case statistically significant (p < 0.05) expression bias was confirmed.

## Discussion

### Masculinization of the zebrafish transcriptome

#### A greater number of male-enriched genes

Our results are consistent with the predictions of the "male sex drive" hypothesis. Three lines of evidence from our study provide reason to believe that gene expression in the zebrafish lineage is "masculinized." First, we discovered a larger total number of male-enriched than female-enriched genes (Table 2), consistent with other animal studies. A recent study, for example, documented this asymmetry in five *Drosophila *species (*D. melanogaster*, *D. simulans*, *D. yakuba*, *D. ananasse*, and *D. virilis*) using species-specific microarrays [12], and additional investigations have reported similar findings in *Drosophila *[11,19,40]. Rin et al. also identified a substantially greater number of male-enriched genes, especially within higher fold change classes, based on a transcriptomic comparison of testis and ovary in mice [14]. In two closely related frog species (*Xenopus laevis *and *X*.*muelleri*), Malone et al. revealed a greater overall number of male-enriched genes and demonstrated an even more pronounced male-biased asymmetry among genes that are also differentially expressed between species [13]. Indeed, others have described a related phenomenon, in which male-enriched genes are greatly overrepresented among groups of genes that demonstrate intra- and inter-specific expression polymorphism, relative to female-enriched and sex-unbiased loci [44-46].

Interestingly, recent studies of sex-biased gene expression in *Danio rerio *have not yielded the same observation of more male-enriched than female-enriched transcripts. In fact, Santos et al. compared ovary and testis transcriptomes in adult zebrafish and reported 1370 male-enriched genes and 1570 female-enriched genes [27], which contrasts with our finding that more genes are male-enriched. One possible source of the discrepancy might be that the experimental animals were treated quite differently in our study. Santos et al. sampled individuals from a "breeding colony" of six males and six females, and histological analysis of experimental ovaries revealed great variation in oogenic stage among individual females [27]. Females in our study spawned on the same day, and were then isolated from males for five days before being sacrificed. Separation of males and females may not reflect conditions zebrafish experience in nature, but our design allowed us to prevent re-mating and standardize reproductive cycles among experimental individuals. Still, a five-day absence of any stimuli produced by the opposite sex might result in significant behavioral and physiological consequences for males and females, and these could explain the differences between the studies. For example, significant changes in gene expression over a very short time period as a consequence of courtship exposure have been documented in *Drosophila *[47]. Additional studies should be conducted to assess the potential for plasticity of sex biases in the transcriptome due to behavioral, environmental, developmental [48,49], and temporal factors.

Differences in array platform and analysis might also explain the discrepancy between studies. Santos et al. employed microarrays constructed from the Sigma-Genosys (Cambridge, UK) Zebrafish OligoLibrary™, which represents approximately the same number of unique transcripts (15,806) as the Affymetrix arrays (14,900), but not necessarily the same transcripts. Furthermore, the expression detection algorithms tailored for Affymetrix GeneChips^® ^are unique, and we applied four of these in this study. It is also worth noting that the microarray fold change estimates from the Santos et al. study are substantially lower (up to 2 orders of magnitude) than the corresponding real-time qPCR fold change estimates, which the authors attribute to spot saturation [27]. Our microarray fold change estimates appear to be more consistent with the real-time qPCR estimates (Table 6), suggesting that array feature saturation is less of a problem in our study. Despite the discrepancy, however, there is agreement between the two studies at the level of expression patterns for individual genes, as nine out of ten top sex-biased genes identified by Santos et al. [27] also appear in our sex-biased gene list (Table 3).

Two other studies addressed sex-biased gene expression in zebrafish, but neither of them is as relevant to this study as the Santos et al. experiment. Wen et al. conducted a whole body male-female comparison of the zebrafish transcriptome using a cDNA microarray representing 8793 unique EST clusters [26]. The authors identified 383 female-enriched genes in their study; however, they make no mention of male-enriched transcripts, and gonads were not analyzed separately. Another microarray study, by Sreenivasan et al., did separate the gonads, in addition to the brain and kidney, from the "rest-of-body," for males and females [28]. They employed cDNA microarrays containing 6370 unique genes derived from zebrafish gonad EST libraries. Sreenivasan et al. reported 881 genes enriched by ≥ 1.5 fold in the testis relative to the common reference control, and 1366 genes enriched by ≥ 1.5 fold in the ovary relative to the common reference control [28]. The report does not provide details regarding the total numbers of male- and female-enriched genes for each organ comparison, so a direct comparison between this study and ours is difficult.

Another surprising result is that we did not identify genes that, according to our strict consensus criteria, demonstrate sex-biased expression at the level of the zebrafish body. A recent study of sex differences with respect to hepatic gene expression, which also utilized the Affymetrix platform, revealed 1249 sex-biased genes (792 male-enriched, 650 female-enriched) in the adult zebrafish liver [30]. Another study, which examined sex differences of the zebrafish brain transcriptome, identified 42 sex-biased genes (18 male-enriched, 24 female-enriched) [29]. This is in stark contrast to Sreenivasan et al. [28], who report 3080 genes as differentially expressed between male and female brains, so it is clear that major differences exist among the other zebrafish studies as well. Our study did not involve a direct organ-to-organ comparison (except for gonads), so it is possible that organ-specific signals of sex-biased gene expression were obscured by background gene expression in other somatic tissues. The lack of sexually dimorphic body gene expression in our study could also be a consequence of high among-individual variance in body gene expression, although we took many steps experimentally to reduce this. Furthermore, our statistical criteria for differentially expressed genes were very conservative, so we likely missed some differentially expressed genes, especially if the differences were small. If we relax our criteria and consider a gene differentially expressed if it appears significant in at least one of the four absolute expression comparisons, then we find 112 body sex-biased genes (78 male-enriched, 34 female-enriched). Of these genes, 26 (9 male-enriched, 17 female-enriched) were consistent with the liver results from Robison et al. [30], but none were consistent with the brain study [29]. The list of 112 genes, and corresponding fold change estimates from the four absolute expression comparisons are included as Additional File 3.

#### Greater expression bias for male-enriched genes

The second pattern indicative of a masculinized transcriptome is an increase in the magnitude of differential expression (i.e. fold change) for male-enriched genes relative to female-enriched genes. Based on our results in *Danio*, male-enriched genes on average demonstrate more extreme sex-biases in expression than female-enriched genes (Fig. 1). This trend was also described by Zhang et al. across seven different *Drosophila *species [12]. If transcript abundance is viewed as a quantitative trait, it becomes apparent that males demonstrate considerably more exaggerated trait values for sex-biased genes than do females. In essence, for traits that are sexually dimorphic (i.e. expression levels of sex-biased genes), males on average appear to demonstrate more extreme phenotypes. This concept should be relevant to an integrated understanding of transcriptomic masculinization, "male-driven" evolution, and sexual dimorphism at additional phenotypic levels.

#### More gonad-soma differences in males

A third result of our study related to reproductive processes and sex-specific gene expression patterns is simply that adult male zebrafish demonstrated many more gonad-soma differences in transcript abundance than females. We detected 5340 genes as differentially expressed between testicular and male body tissue (3002 testis-upregulated, 2338 testis-downregulated). In comparison, only 2380 genes were identified as being differentially expressed between ovarian and female body tissue (981 ovary-upregulated, 1399 ovary-downregulated). These striking transcriptional differences at a tissue-specific level are likely reflections of fundamental reproductive differences between males and females. A microarray study of *D. melanogaster *adults revealed a similar sex disparity in gonad-biased gene expression and also reported that the expression magnitude of testis-upregulated genes is substantially greater than that for ovary-upregulated genes [40]. Because none of the 981 ovary-upregulated genes identified in our study demonstrated fold change values greater than four, whereas fold change values for 554 testis-enriched genes exceeded six, zebrafish may also conform to this pattern. A general interpretation of this trend might be that there are more specific transcripts essential to processes that take place in the testes, relative to specific transcripts in ovarian tissue.

A small comparison of testis-upregulated or testis-specific genes from other zebrafish studies [28,42] to those identified as testis-upregulated in our study indicates a high level of agreement (see "testis-upregulated" section of Table 3). In contrast, many of the top ovary-specific or ovary-upregulated genes identified consistently in these studies are absent from our list of top ovary-upregulated genes (Table 5). Why our study differs from the others in this respect remains an open question. Again, the fact that we separated males from females five days prior to sample collection may partially explain the discrepancy, especially if females experience major changes in hormone profiles in the absence of males. High body gene expression variance among females in our samples could also explain why ovary-upregulated genes from the other studies did not demonstrate statistically different expression levels in our study. Additional File 7, a more detailed version of Table 3, includes ten reportedly ovary-upregulated genes and the relevant expression value means, standard errors, and fold change estimates from our data set.

A particularly important class of female reproductive genes, which correspond to members of the zona pellucida egg coat glycoprotein superfamily, demonstrate ovary-specific expression patterns according to several zebrafish studies (*zp1 *[43]; *zp2 *[43,50]; *zp3 *[50,51]). We, however, identified none of the zona pellucida homologs represented on the zebrafish GeneChip^® ^as significantly ovary-upregulated (See Additional file 8 for a list of *zp *genes, expression value means, and standard errors for each absolute expression analysis). This result is surprising, and the expression values in Additional file 8 indicate high female body *zp *expression in addition to expectedly strong expression in ovaries. Contamination of the body sample with ovarian tissue could produce this result but is unlikely since we completely removed all visible ovarian tissue from each individual. Even if a dissection left as much as half of the total ovarian tissue inside a body sample, one would not expect equal or greater body transcript abundance (for a truly ovary-upregulated gene), because the contaminating ovary signal would be greatly diluted by the female body RNA. Furthermore, if the female body samples were contaminated with ovarian tissue, we would expect many false positives with respect to male and female body differences, which is clearly not the case. We, therefore, maintain that high female body *zp *expression in our experiment is either real or a reflection of problematic *zp *array probesets. In general, there seems to be some disagreement across studies with respect to tissue specific patterns of *zp *gene expression. For example, significant expression of *zp1 *and *zp2 *has been documented in ovary-excised females [26], and expression of *zp3 *in female skeletal muscle has also been described [43]. Furthermore, a recent study (which also used Affymetrix zebrafish arrays) of sex-biased gene expression in the liver of zebrafish reported that *zp2.2*, *zp3*, *zp3a.1*, *zp3b*, and *zpcx *are all expressed at high levels and are all female-enriched [30]. Based on an estimate by Liu et al., there are likely 10 - 15 *zp2 *and 17 - 21 *zp3 *paralogs alone distributed throughout the zebrafish genome [52], so assaying expression of individual paralogs may not be as straightforward as is assumed. We cannot say for certain that our results reflect this specific problem, but across-study differences in *zp *probe composition might explain some of the inconsistencies in tissue-specific expression patterns of zona pellucida genes.

### Genomic differences and sex-biased gene expression

In the absence of dosage compensation, having two copies of a sex chromosome (i.e. the homogametic sex) could allow increased expression of sex chromosome genes in the homogametic sex relative to the heterogametic sex [53]. This is not likely the reason for sex-biased gene expression in zebrafish, however, because karyotypes of the *Danio rerio *genome fail to reveal heteromorphic sex chromosomes [54]. Furthermore, no sex-linked genetic markers or key sex-determination loci have been described in zebrafish as of the completion of our study [20,55]. This suggests that sexually dimorphic gene expression and sexual dimorphism are not explained solely or directly by genome differences between male and female zebrafish. A more plausible scenario is that environmental or genetic conditions initiate sexual differentiation, followed by hormonal differences which cascade into large scale sex-biased gene expression and ultimately into other phenotypic aspects of sexual dimorphism, such as morphological and behavioural differences.

### The evolution of sex-biased gene expression

Our study does not specifically address mechanisms potentially responsible for the adaptive evolution of sexually dimorphic gene expression, but these are worth considering here briefly. In general, two processes are capable of generating selection for differential transcript abundance in males and females. Sexual selection could drive the evolution of transcript abundance via mating or fertilization advantages to individuals within a population. Because the general intensity of sexual selection may be different between the sexes [56], it could generate an antecedent for different adaptive trajectories between males and females. Similarly, sex-specific ecological selection could drive the evolution of gene expression via survival, fecundity, or fertility advantages to members of one or the other sex. If there is intrinsic sex-limitation of the novel transcript abundance from the outset, owing to existing sex-differences in genetic background for example, sexual selection or sex-specific ecological selection can automatically result in sexual dimorphism. If not, a secondary mechanism such as intersexual conflict is required to reinforce stable sexual dimorphism in transcript abundance. Under this scenario, a transition to the male- or female- selected expression "optimum" is constrained, due to a different optimum in the opposite sex. This process generates selection for sex-limited gene expression, and sexually dimorphic expression is a possible response.

Few attempts have been made to rigorously test which (if any) of these processes are responsible for the great degree of sex-biased gene expression observed across animal taxa, but work by Connallon and Knowles [57] suggests a signature of sexual conflict in *Drosophila *sex-biased gene expression patterns. Sexual selection in zebrafish has not been quantified formally, but the species exhibits little morphological sexual dimorphism, and observations of mating patterns suggest conditionally high variance in male and female mating success [25]. More extensive studies comparing gene expression patterns among closely related species that differ with respect to the above selective forces will become feasible in the wake of advancing genomics resources for non-model organisms, and this should greatly improve our evolutionary understanding of sex-biased gene expression.

## Conclusion

In general, our microarray results suggest that adult zebrafish demonstrate sexually dimorphic gene expression profiles across a large proportion of the genome. We detected a greater abundance of male- than female-enriched genes, and found that male-enriched genes demonstrate higher fold changes on average than female-enriched genes. Male zebrafish also demonstrated many more expression differences between body and gonads than did females. These findings are consistent with male-biased patterns of gene expression described in studies of other animal taxa, although they are at odds in some ways with recent zebrafish studies. The discrepancies are discussed, but identifying their sources is difficult due to very different objectives, analyses, and experimental approaches across studies. Sex-biases in gene expression deserve attention because they may explain important differences between males and females, an extension of the realization that gene regulation plays a major role in phenotypic evolution.

## Authors' contributions

CMS, GEC, and AGJ, conceived the study and drafted the manuscript. All analyses were conducted at Texas A&M University, and all authors participated in data analysis. CMS prepared the samples and synthesized the analyses. All authors read and approved the final manuscript.

## Supplementary Material

Additional file 1 **Annotation information and expression rankings for all male-enriched genes**. Microsoft Excel^® ^spreadsheet containing all male-enriched GeneChip^® ^probe sets, gene identifiers, fold change rank for each absolute expression analysis, overall mean rank, and annotation details if available. Click here for file

Additional file 2 **Annotation information and expression rankings for all female-enriched genes**. Microsoft Excel^® ^spreadsheet containing all female-enriched GeneChip^® ^probe sets, gene identifiers, fold change rank for each absolute expression analysis, overall mean rank, and annotation details if available. Click here for file

Additional file 3 **Genes potentially expressed differentially between male and female body**. Microsoft Excel^® ^spreadsheet containing sex-biased genes (body) significant (FDR = 0.05) in at least one absolute expression comparison, and relevant fold change estimates. Click here for file

Additional file 4 **Annotation information and expression rankings for all testis-upregulated genes**. Microsoft Excel^® ^spreadsheet containing all testis-upregulated GeneChip^® ^probe sets, gene identifiers, fold change rank for each absolute expression analysis, overall mean rank, and annotation details if available. Click here for file

Additional file 5 **Annotation information and expression rankings for all ovary-upregulated genes**. Microsoft Excel^® ^spreadsheet containing all ovary-upregulated GeneChip^® ^probe sets, gene identifiers, fold change rank for each absolute expression analysis, overall mean rank, and annotation details if available. Click here for file

Additional file 6 **Real-time qPCR data**. Microsoft Excel^® ^spreadsheet containing original qPCR expression values, relevant calculations, and statistical test details. Click here for file

Additional file 7 **Detailed across-study comparison of sex- and gonad-biased gene expression in zebrafish**. Microsoft Excel^® ^spreadsheet containing the genes listed in Table 3, plus relevant expression means, standard errors, and fold change estimates for each of the four absolute expression comparisons. Click here for file

Additional file 8 **Zona pellucida expression data**. Microsoft Excel^® ^spreadsheet containing zona pellucida genes represented in this experiment, plus relevant expression means and standard errors for each of the four absolute expression comparisons. Click here for file
